# Tolerogenic Nanoparticles Induce Antigen-Specific Regulatory T Cells and Provide Therapeutic Efficacy and Transferrable Tolerance against Experimental Autoimmune Encephalomyelitis

**DOI:** 10.3389/fimmu.2018.00281

**Published:** 2018-03-02

**Authors:** Robert A. LaMothe, Pallavi N. Kolte, Trinh Vo, Joseph D. Ferrari, Tracy C. Gelsinger, Jodie Wong, Victor T. Chan, Sinthia Ahmed, Aditi Srinivasan, Patrick Deitemeyer, Roberto A. Maldonado, Takashi K. Kishimoto

**Affiliations:** ^1^Selecta Biosciences Inc., Watertown, MA, United States

**Keywords:** nanoparticles, immunological tolerance, rapamycin, regulatory T cells, experimental autoimmune encephalomyelitis

## Abstract

T cells reacting to self-components can promote tissue damage when escaping tolerogenic control mechanisms which may result in autoimmune disease. The current treatments for these disorders are not antigen (Ag) specific and can compromise host immunity through chronic suppression. We have previously demonstrated that co-administration of encapsulated or free Ag with tolerogenic nanoparticles (tNPs) comprised of biodegradable polymers that encapsulate rapamycin are capable of inhibiting Ag-specific transgenic T cell proliferation and inducing Ag-specific regulatory T cells (Tregs). Here, we further show that tNPs can trigger the expansion of endogenous Tregs specific to a target Ag. The proportion of Ag-specific Treg to total Ag-specific T cells remains constant even after subsequent Ag challenge in combination with a potent TLR7/8 agonist or complete Freund’s adjuvant. tNP-treated mice do not develop experimental autoimmune encephalomyelitis (EAE) after adoptive transfer of encephalitogenic T cells; furthermore, tNP treatment provided therapeutic protection in relapsing EAE that was transferred to naïve animals. These findings describe a potent therapy to expand Ag-specific Tregs *in vivo* and suppress T cell-mediated autoimmunity.

## Introduction

Maintenance of peripheral immunological tolerance is a dynamic and continuous process. Most self-reactive T lymphocytes are deleted in the thymus or differentiate into natural T regulatory cells (Tregs), but some can also enter the pool of naive circulating cells. Self-reactive naive T cells that escape the thymus and encounter their cognate antigen (Ag) in the periphery can differentiate into induced Tregs (1, 2). Tregs maintain immune homeostasis *in vivo*, and their dysfunction, caused by the loss of expression of the master transcription factor Foxp3, leads to the development of immunodysregulation polyendocrinopathy enteropathy X-linked syndrome (IPEX) in humans characterized by systemic T cell activation and multiorgan autoimmunity (3). In most autoimmune diseases, genetic and environmental factors result in the dysregulated expansion of autoreactive lymphocytes that mediate damage to self-tissue (4, 5). For example, in multiple sclerosis, a chronic neuroinflammatory disease, myelin proteins are actively targeted by immune cells resulting in myelin degradation, loss of neuronal function, and progressive paralysis (6). There has been substantial progress in the identification of small molecule and biological therapies that ameliorate disease, but there is no cure (7, 8).

Antigen-specific immune tolerance has been a long-standing goal in the treatment of autoimmune diseases. Dendritic cells (DCs) and other Ag-presenting cells are at the crossroads of immunity and tolerance. The context in which DCs encounter Ag can determine the nature of the T cell response (9). Danger signals, such as pathogen-associated molecular patterns (PAMPs) or damage-associated molecular patterns (DAMPs), induce DC maturation resulting in the expression of co-stimulatory molecules and cytokines that drive effector T cell activation and differentiation (10). Vaccines often employ an adjuvant to provide this “danger” context to induce an adaptive, Ag-specific effector response (11). Recently, there has been interest in the identification of “tolerogenic adjuvants” that would enable the induction of Ag-specific Tregs rather than effector T cells (12). Rapamycin, an inhibitor of the mTOR signaling pathway, has been shown to induce tolerogenic DCs (itDCs) *in vitro*, which are capable of inducing regulatory T cells and suppressing disease when adoptively transferred *in vivo* (13–15). Our group and others have demonstrated that tolerogenic nanoparticles (tNPs; also known as synthetic vaccine particles or SVPs) and microparticles encapsulating rapamycin induced tolerogenic DCs *in vivo* causing the differentiation of Ag-specific regulatory T cells (16–20).

In this study, we further characterize the induction of Ag-specific endogenous Tregs by acute treatment with tNPs composed of polylactic acid (PLA) and poly(lactic-co-glycolic acid) (PLGA) polymers encapsulating peptide Ag and rapamycin. We demonstrate therapeutic efficacy of tNPs in a model of relapsing experimental autoimmune encephalomyelitis (rEAE) and show that tolerance can be adoptively transferred from a tNP-treated animal to a naive animal. Furthermore, mice treated with tNPs were protected against EAE following transfer of encephalitogenic T cells.

## Materials and Methods

### Mouse Models

The following animals were used: female C57BL/6nTac (RRID:IMSR_TAC:b6), B6.Cg-Tg(TcraTcrb)425Cbn/J (RRID:IMSR_JAX:004194), B6.129S6-*Rag2^tm1Fwa^* N12 (RRID:IMSR_TAC:1329), B6.SJL-*Ptprc^a^*/BoyAiTac (RRID:IMSR_TAC:4007), and SJL/J (RRID:IMSR_JAX:000686). Experiments involving animals were performed in compliance with state and federal regulations and approved by the Institutional Animal Care and Use Committee of Selecta Biosciences or Hooke Laboratories.

### Nanoparticles (NPs)

Manufacture of NPs has been previously described (18). PLGA, pegylated PLA (PLA-PEG), and rapamycin were dissolved in dichloromethane to form the oil phase. An aqueous solution of Ag (OVA_323–339_ peptide, 2W1S peptide, or PLP_139–151_ peptide) was then added to the oil phase and emulsified by sonication (Branson Digital Sonifier 250A). Following emulsification of the Ag solution into the oil phase, a double emulsion was created by adding an aqueous solution of polyvinylalcohol and sonicating a second time. The double emulsion was added to a beaker containing phosphate buffer solution and stirred at room temperature for 2 h to allow the dichloromethane to evaporate. When creating NPs containing rapamycin but no Ag, or NPs without any encapsulated agents, a similar oil-in-water single-emulsion process was used. The resulting NPs were washed twice by centrifuging at 75,600 × *g* and 4°C followed by resuspension of the pellet in phosphate-buffered saline (PBS). MHC class II (MHCII) peptides used were 2W1S (2W, EAWGALANWAVDSA, CSBio), OVA_323-339_ (OVA_323_, ISQAVHAAHAEINEAGR, Bachem B06481), or PLP_139-151_ (PLP_139_, HCLGKWLGHPDKF, Genemed Synthesis). NPs containing peptide alone are denoted as follows: NP[2W], NP[OVA_323_], and NP[PLP_139_]. NPs containing peptide and rapamycin are denoted as follows: NP[2W-Rapa], NP[OVA_323_-Rapa], and NP[PLP_139_-Rapa]. NPs containing peptide and rapamycin are referred herein as tNPs. Empty NPs (NP[Empty]) were used as controls.

### EAE Models

Relapsing EAE was induced by injection of SJL mice subcutaneously (s.c.) at four sites in the back with PLP_139_ emulsified in complete Freund’s adjuvant (CFA) followed by intraperitoneal (i.p.) injection of 154ng of pertussis toxin (PTx) 2 h later (Hooke Laboratories EK-2120). Pathogenic cells used for adoptive transfer models of EAE were propagated by immunizing SJL mice with PLP_139_/CFA (Hooke Laboratories EK-0120). Seven days later, spleens were excised from immunized mice and single-cell splenocyte suspensions were isolated through mechanical dissociation. Red blood cells were lysed (Sigma R7757) and splenocytes were restimulated *in vitro* in RPMI 1640 containing HEPES (Life Technologies 15630080), l-glutamine–penicillin–streptomycin (Sigma G6784), MEM Non-Essential Amino Acids Solution (Life Technologies 11140-050), MEM Sodium Pyruvate Solution (Life Technologies 11360-070), and 2-Mercaptoethanol (1000X, Life Technologies 21985-023) with Hooke PLP_139_ in TC Media, 100× (Hooke Labs DS-0121) for 3 days before being injected i.p. into recipient mice. Regulatory cell adoptive transfer studies were carried out in a similar manner. After s.c. treatment of donor mice with NPs, their spleens were excised, and single-cell splenocyte suspensions were isolated through mechanical dissociation. *In vitro* culture was carried out as done with pathogenic cells with the modification that splenocytes were restimulated with PLP_139_ in the presence of 100 U/ml IL-2. Sickness scoring assessments were carried out as previously described (18). EAE was scored on a 0–5 scale as follows: 0, no obvious changes in motor functions of the mouse in comparison with non-immunized mice; 1, limp tail; 2, limp tail and weakness of hind legs; 3, limp tail and complete paralysis of hind legs (most common) or limp tail with paralysis of one front and one hind leg; 4, complete hind leg and partial front leg paralysis; 5, death or euthanized because of severe paralysis. Demyelination was scored by H&E staining of central nervous system (CNS) sections with the NP[Empty] group used as the baseline for tissue disruption.

### Immunizations and Treatments

100µg of 2W peptide admixed with 20µg R848 (Selecta Biosciences) or emulsified 1:1 with CFA (Sigma F5881) was injected i.p. or s.c. as an immunization. NPs containing peptide alone were injected at a 4–5µg dose of peptide i.v. or s.c. NPs containing rapamycin alone (NP[Rapa]) were injected at a 50µg dose of rapamycin i.v. or s.c. tNPs were injected at a 4 to 5µg dose of peptide and a 50µg dose of rapamycin i.v. or s.c.

### Endogenous 2W1S:IA^b^ + T Cell Enrichment

2W1S-specific CD4 T cells were enriched and enumerated as previously described (21). Briefly, mice were sacrificed, and splenocytes were isolated by mechanical dissociation. 2W1S:IA^b^ tetramers conjugated to allophycocyanin (APC) or phycoerythrin (PE) (NIH Tetramer Core Facility, mouse 2W1S) were incubated at room temperature with splenocytes for 45 min. Cells were washed then incubated with anti-APC or anti-PE microbeads (Miltenyi Biotec, 130-090-855, 130-048-801) for 20 min at 4^o^C. Cells were washed, resuspended, and eluted over a magnetized bead-packed LS column (Miltenyi Biotec, 130-042-401). Positively selected 2W1S:IA^b^ cells were expelled from columns and phenotyped by flow cytometry.

### Flow Cytometry

Samples were analyzed on a Becton Dickinson FACSCanto II with the following conjugated antibodies: TCRβ (BD Biosciences 553171, RRID:AB_394683), CD45R (BD Biosciences 561226, RRID:AB_10563910), CD45.2 (BioLegend 109830, RRID:AB_1186098), CD4 (BioLegend 100433, RRID:AB_893330), CD44 (BioLegend 103029, RRID:AB_830786), CD11b (BioLegend 101245, RRID:AB_2561390), CD11c (BioLegend, 117338, RRID:AB_2562016), Foxp3 (Thermo Fisher 12-5773-82, RRID:AB_465936), TCRVa2 (Thermo Fisher 17-5812-82, RRID:AB_1659733), and Live/Dead Fixable Viability Stain Aqua (Thermo L34957).

### IFNγ ELISpot

Sterile, white 96-well filter plates with 0.45- µm pore size Hydrophobic PVDF membrane (EMD Millipore, Billerica, MA, USA, Cat#MSIPS4W10) were coated with 5µg/ml of purified anti-mouse IFNγ capture antibody (BD Pharmingen 551881) in dilution buffer (DB) (PBS 1× Corning cellgro 21-040CV) with 2% fetal bovine serum (FBS; heat inactivated, Medsupply partners MSP-1003-3/Hi) overnight at 4°C. Unbound antibodies were discarded by emptying the wells followed by blocking with complete medium (CM) [RPMI 1640 (1× with Corning glutagro, Mediatech 10-104-CV) supplemented with FBS (FBS, heat inactivated, Medsupply partners MSP-1003-3/Hi), Pen/Strep and l-glutamine (100×), Gibco by Life technologies 10378-016, 2-Mercaptoethanol (55 mM), Gibco by Life technologies 21985] for 90 min at 37°C. 1 × 10^5^ lymph node cells per well were incubated in the IFNγ capture antibody-coated plates in CM as a test condition overnight at 37°C. The plates were washed three times with wash buffer (WB) [PBS containing 0.05% Tween20 (Sigma P1379)] after which the plates were incubated for 90 min at room temperature with 2µg/ml biotinylated anti-mouse IFNγ detection antibody (BD Pharmingen 551881) diluted in DB. The plates were washed three times with WB followed by Stretavidin-HRP (BD Pharmingen 557630) addition diluted 1:100 in DB and incubated for 1 h at room temperature. The plates were washed three times with WB and three times with PBS followed by addition of AEC substrate (BD Pharmingen, 551951) for spot development. The plates were read on a Zeiss KS Elispot reader system, using KS Elispot software version 4.9.16.

## Results

### tNPs Inhibit the Proliferation of Ag-Specific Effector CD4^+^ T Cells

**Figure 1:**
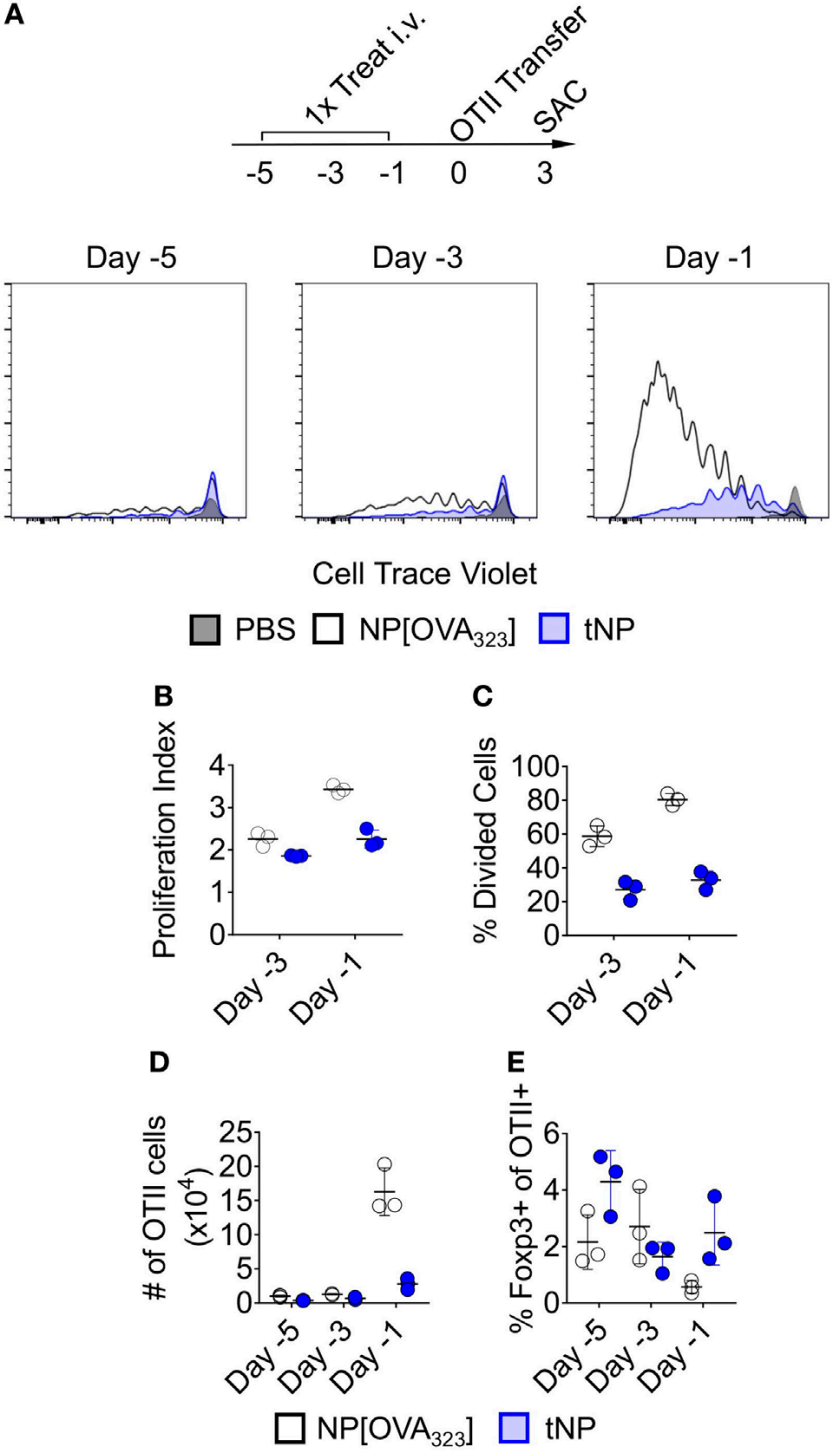
Tolerogenic nanoparticles (tNPs) inhibit the proliferation of antigen (Ag)-specific effector CD4^+^ T cells. **(A–E)** Rag2^−/−^ mice were treated i.v. with PBS, NP[OVA_323_] at a 5µg dose of OVA_323_ peptide, or tNP (NP[OVA_323_-Rapa]) at a 5µg dose of OVA_323_ peptide and a 50µg dose of rapamycin on days −5, −3, or −1. OTII cells were transferred to treated Rag^−/−^ mice on day 0 and their spleens assayed for OTII cells on day 3. **(A)** Cell Trace Violet flow cytometry histograms of TCRVα2^+^ CD4^+^ gated OTII splenocytes, **(B)** OTII proliferation index, **(C)** % divided cells, **(D)** total cell number (#), and **(E)** % Foxp3 expression. The results represent an *N* = 3 from one experiment.

We evaluated the activation of OTII transgenic T cells that recognize the 323–339 peptide of chicken ovalbumin (OVA_323_) after adoptive transfer into mice that were previously treated with NPs containing the OVA_323_ peptide alone (NP[OVA_323_]) or tNPs containing both OVA_323_ and rapamycin. Rag2^−/−^-recipient mice were used to ensure that no endogenous lymphocytes would compete with the OTII T cells for binding to OVA_323_ peptide presented by Ag-presenting cells. NP[OVA_323_] or tNPs were administered to the recipient animals 1, 3, or 5 days before OTII CD4^+^ T cell transfer (Figure 1A). Recipient animals were sacrificed 3 days after cell transfer, and splenic OTII T cells were assayed for cell proliferation and Foxp3 expression. No differences in total numbers of OTII cells were observed when NPs were administered 3 and 5 days prior to cell transfer (Figure 1D); however, reduced proliferation capacity, a reduction in the total numbers of OTII cells and an increase in the proportion of Foxp3^+^ OTII T cells were observed after tNP treatment administered 1 day prior to cell transfer compared to treatment with NP[OVA_323_] (Figures 1B–E). These results corroborate our previous findings showing a greater proportion of Foxp3^+^ Tregs after tNP treatment compared to NP[OVA_323_] treatment. A smaller proportion of OTII T cells enter division after tNP treatment compared to NP[OVA_323_], and the extent of cell proliferation was diminished.

### tNPs Increase the Total Number and Proportion of Endogenous T Cells Expressing Foxp3 in an Ag-Specific Fashion

**Figure 2:**
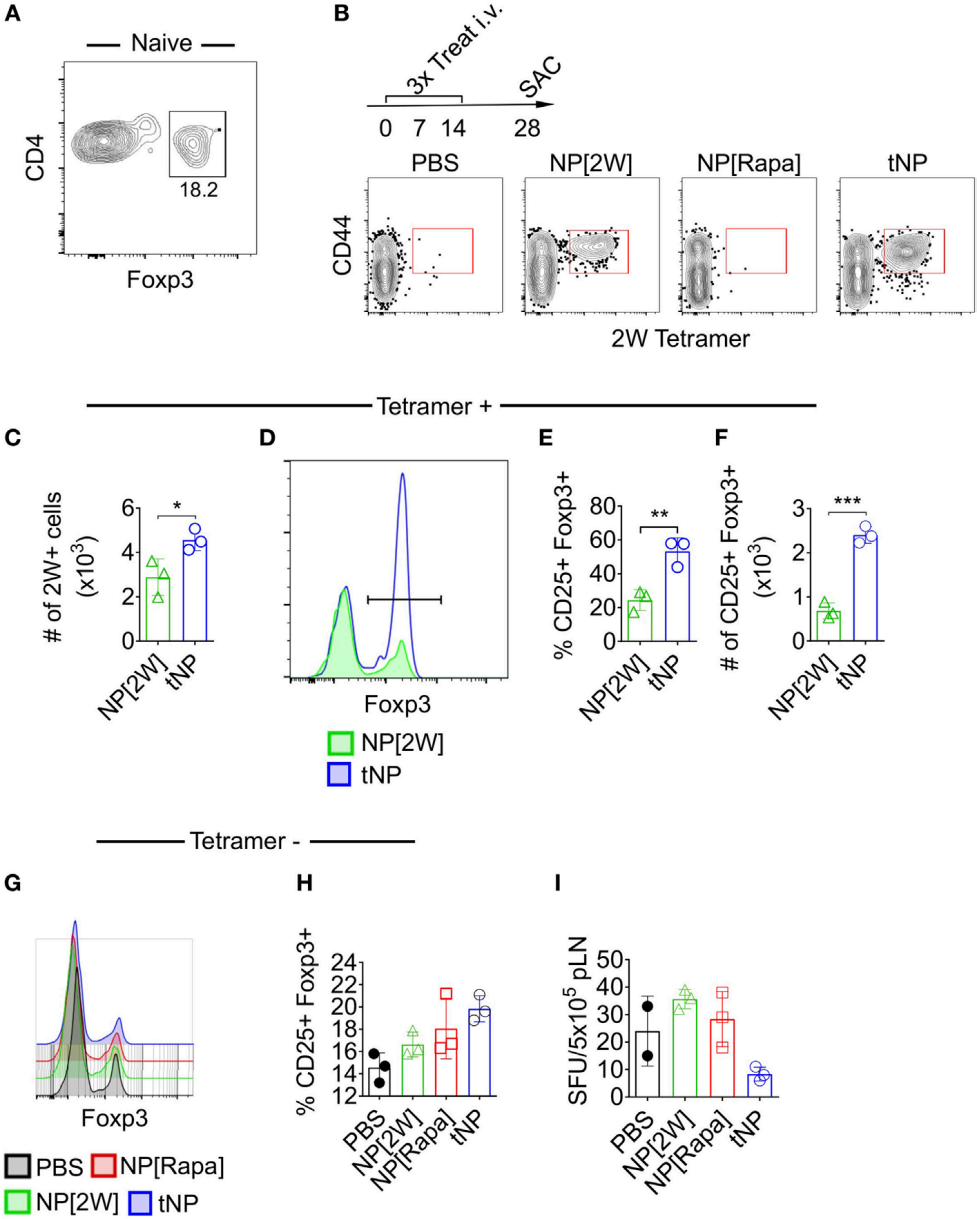
Tolerogenic nanoparticles (tNPs) increase the total number and proportion of endogenous T cells expressing Foxp3^+^ in an antigen-specific fashion. **(A)** Splenocytes from five naive mice were pooled and assayed with 2W:Ia^b^ tetramers after MACS enrichment to identify 2W-specific CD4 T cells (2W^+^CD4^+^). Their expression of Foxp3 was quantified. **(B)** WT mice were treated with PBS, NP[2W] at a 4µg dose of 2W peptide, nanoparticles containing rapamycin alone (NP[Rapa]) at a 50µg dose of rapamycin, or tNP (NP[2W-Rapa]) at a 4µg dose of 2W peptide and 50µg dose of rapamycin on days 0, 7, and 14, and their spleens were assayed with 2W:Ia^b^ tetramers to identify 2W^+^CD4^+^ cells. **(B)** Dot plots of 2W:Ia^b^-PE MACS-enriched splenocytes stained as live/dead^−^, CD11b^−^, CD11c^−^, B220^−^, CD4^+^. **(C)** # of 2W:Ia^b^ tetramer-positive cells from NP[2W] and tNP-treated groups. **(D)** Expression of Foxp3^+^ on 2W^+^CD4^+^ T cells. **(E)** % and **(F)** # of 2W^+^CD4^+^ cells that are Foxp3^+^. **(G)** Expression of Foxp3^+^ on 2W-CD4^+^ T cells. **(H)** % of 2W-CD4^+^ cells that are Foxp3^+^. **(I)** # of IFNγ spot-forming units (SFUs) from draining lymph nodes of mice treated and challenged 2W/R848 subcutaneously in **(B)**. Error bars indicate SD. The results from **(C–F)** represent an *N* = 3 from one experiment of two representative experiments. The results from **(A,B,G–I)** represent an *N* = 3 from one experiment. Statistics are derived from a one-way ANOVA with Tukey Multiple Comparison test for **(C,E,F)**. Significance: *p < 0.05; **p < 0.005; ***p < 0.0005.

Next we utilized MHCII tetramers loaded with the 2W1S (2W) peptide to evaluate the effects of tNPs on endogenous wild-type Ag-specific CD4^+^ T cells. This tetramer system has been validated to study T helper cell responses (22), T follicular helper cell differentiation (23), and Treg-mediated tolerance (24). We queried 2W:MHCII^+^ endogenous cells from naive mice and found 18.2% were Foxp3 positive (Figure 2A). This served as our baseline proportion of 2W-specific Foxp3^+^ cells in naive animals. We then compared this percentage to that from mice treated i.v. with three weekly injections of PBS, NPs containing 2W peptide alone (NP[2W]), NP[Rapa], or tNPs containing 2W peptide and rapamycin (Figure 2B). Two weeks following treatment, all mice were challenged i.v. with 50µg free 2W peptide, and their splenocytes were assayed for 2W-specific CD4 T cells 2 h after challenge. MACS-enriched 2W-specific T cells were gated *via* the following gating scheme: Live/Dead Fixable Aqua^−^, B220^−^, CD11c^−^ CD11b^−^ CD4^+^, CD44^+^, 2W:MHCII^+^, and then probed for Foxp3 expression (Figure 2B). When comparing across all groups, only those mice treated with tNP or NP[2W] showed a significant proliferation of 2W-specific CD4^+^ T cells (Figure 2B). The total number of 2W-specific cells was significantly higher with tNP treatment compared to NP[2W] alone (Figure 2C). This result indicates that neither NP[2W] nor tNP treatment caused depletion of Ag-specific CD4 T cells. Importantly, the proportion and total number of 2W-specific Foxp3^+^CD4^+^ cells was significantly higher in the tNP-treated group compared to NP[2W] treatment alone (Figures 2D–F). This level of increase in Foxp3^+^ cells was specific to 2W:MHCII^+^CD4^+^ T cells, as it was not observed within the 2W:MHCII-negative population of CD4^+^ T cells (Figures 2G,H). These results indicate that tNP treatment selectively increased Ag-specific endogenous Tregs. In contrast, there was no difference in the proportion of 2W-specific Foxp3^+^ T cells between the naive and NP[2W]-treated animals. Another cohort of tNP-treated mice also had fewer IFNγ spot-forming units (SFUs) from draining lymph node cells than NP[2W]-treated animals 5 days after s.c. challenge with 2W and R848, a TLR7/8 agonist (Figure 2I). Neither NP[2W] nor tNP treatment induced Tbet, Gata3, IFNγ, or IL-4 in 2W-specific CD4^+^ T cells after peptide restimulation *in vivo* (Figure S1 in Supplementary Material).

### tNP Treatment Increases Ag-Specific Endogenous Foxp3^+^ Tregs That Withstand Ag Challenge in the Presence of a TLR Agonist or CFA

**Figure 3:**
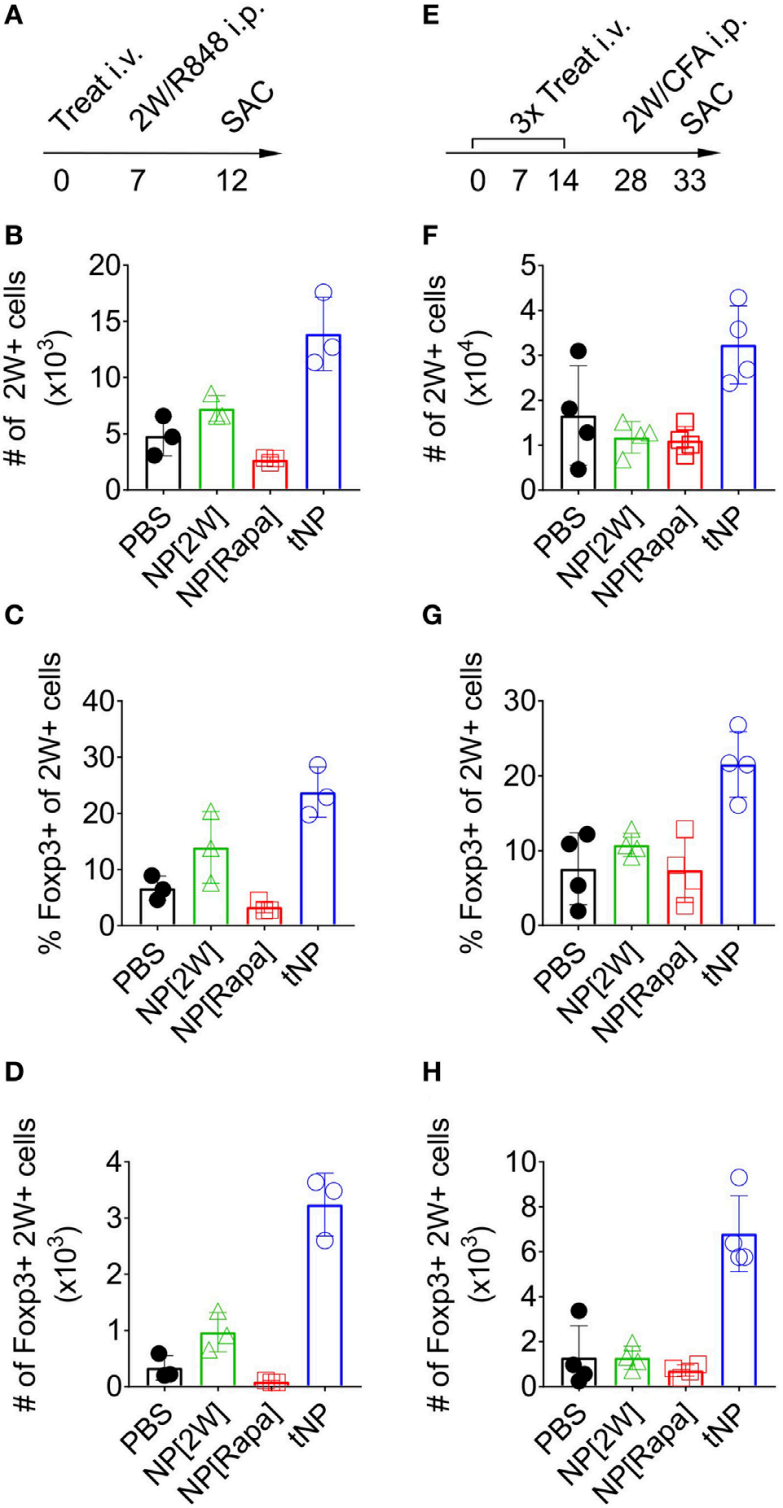
Tolerogenic nanoparticle (tNP) treatment increases antigen-specific endogenous Foxp3^+^ Tregs that withstand antigen challenge in the presence of a TLR agonist or complete Freund’s adjuvant (CFA). **(A)** WT mice were treated once i.v on day 0 with PBS, NP[2W] at a 5µg dose of 2W peptide, nanoparticles containing rapamycin alone (NP[Rapa]) at a 50µg dose of rapamycin and tNP at a 5µg dose of 2W peptide and 50µg dose of rapamycin. Mice were then challenged i.p. with 100µg 2W peptide and 20µg R848 on day 7, and spleens were assessed for 2W^+^CD4^+^ T cells on day 12. MACS-enriched 2W^+^ cells were Live/Dead Fixable Aqua^−^, B220^−^, CD11c^−^, CD11b^−^, CD4^+^, CD44^+^, 2W:Ia^b+^ and then probed for Foxp3 expression. **(B)** Total numbers (#) of 2W^+^CD4^+^ T cells from the spleen. **(C)** % and **(D)** total # of 2W^+^CD4^+^ T cells that are Foxp3^+^. **(E)** WT mice were treated three times weekly, i.v. on days 0, 7, and 14 with PBS, NP[2W] at a 5µg dose of 2W peptide, NP[Rapa] at a 50µg dose of rapamycin, and tNP at a 5µg dose of 2W peptide and 50µg dose of rapamycin. Mice were then challenged i.p. with 100µg 2W peptide admixed 1:1 with CFA on day 28, and spleens were assessed for 2W^+^CD4^+^ T cells on day 33. **(F)** total # of 2W^+^CD4^+^ cells from the spleen. **(G)** % and **(H)** total # of 2W^+^CD4^+^ cells that are Foxp3^+^. The results from **(B–D)** represent an *N* = 3 from one experiment. The results from **(F–H)** represent an *N* = 4 from one experiment.

To assess the stability of Foxp3 expression on endogenous 2W-specific CD4 T cells, mice were injected i.p. with 100μg of 2W peptide admixed with 20μg of R848, a potent TLR7/8 agonist (Figure 3A). A single tNP treatment administered before challenge induced an increase in the total number of 2W-specific CD4^+^ T cells compared to PBS and single-component NP controls (NP[2W] and NP[Rapa], Figure 3B). The average proportion of 2W-specific Foxp3^+^CD4^+^ T cells within the 2W:MHCII^+^ population was higher in the tNP-treated (24%) and NP[2W]-treated (14%) groups compared to PBS and NP[Rapa]-treated controls (6.6 and 3.3%, respectively) (Figure 3C). However, the total number of 2W-specific Foxp3^+^CD4^+^ T cells found after tNP treatment (3.2 × 10^3^ cells) was at least ninefold higher compared to all other groups (0.34 × 10^3^, 0.96 × 10^3^, and 0.09 × 10^3^ cells in PBS, NP[2W], and NP[Rapa] treated animals, respectively) (Figure 3D). These results suggest that the expression of Foxp3 in Ag-specific endogenous CD4 T cells after tNP treatment was increased compared to all single-component NP controls, even after Ag challenge in the presence of a potent TLR agonist. Similar results were observed in a three treatment model followed by challenge with Ag in CFA (Figure 3E). The total number of 2W-specific CD4^+^ T cells (3.23 × 10^4^) along with the proportion (21.5%) and total number of Foxp3^+^2W^+^CD4^+^ T cells (6.8 × 10^3^) was higher in mice treated with tNP compared to all other single-component NP controls (Figures 3F–H). The increased proportion and total number of 2W-specific Foxp3^+^ T cells, compared to PBS controls, was only observed in mice that received tNP alone (Figures 2E,F) or tNP followed by Ag challenge (Figures 3G,H). Treatment with NP[2W] did not show an increase in the proportion or number of 2W-specific Foxp3^+^CD4^+^ T cells compared to PBS controls after challenge (Figures 3G,H). Neither one nor three NP[Rapa]-alone treatments increased Ag-specific Treg numbers above PBS controls (Figures 3C,D,G,H). These results further underscore the Ag-specific and pro-tolerogenic nature of tNP treatment.

### tNP Treatment Confers Therapeutic Efficacy in a Relapsing Remitting Model of Experimental Autoimmune Encephalomyelitis (rEAE)

**Figure 4:**
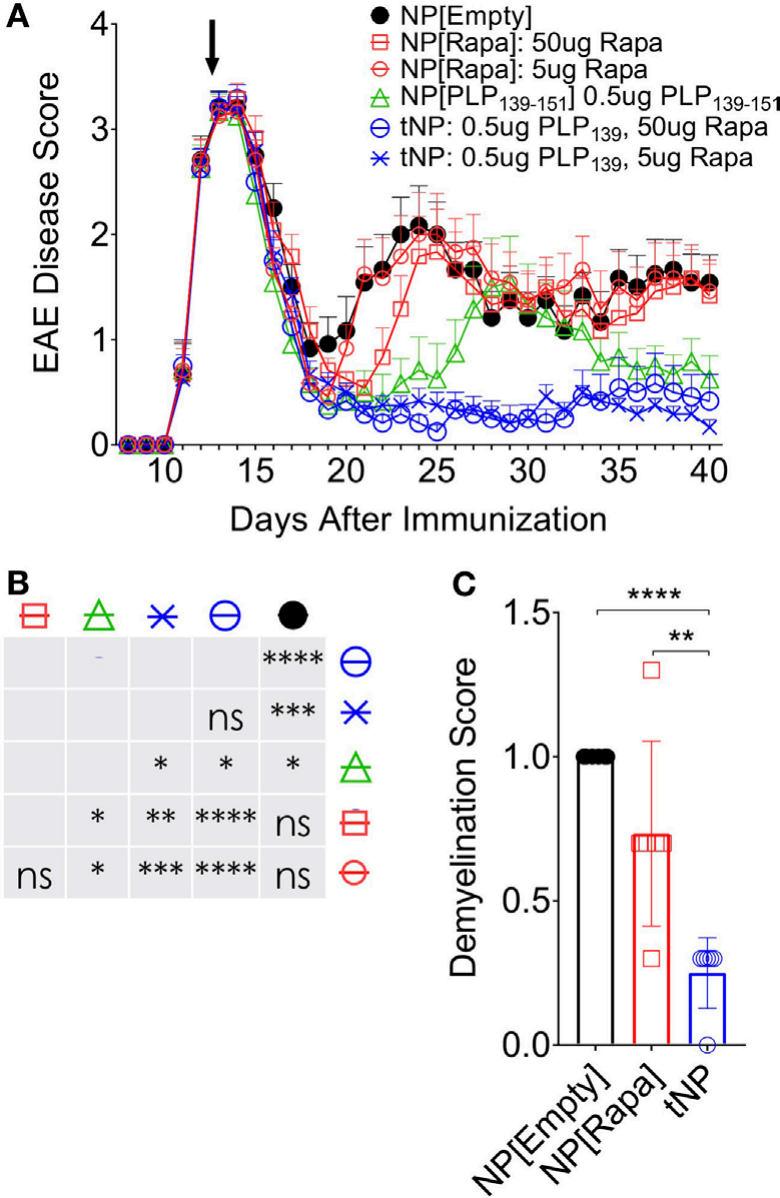
Tolerogenic nanoparticle (tNP) treatment confers therapeutic efficacy in a model of relapsing experimental autoimmune encephalomyelitis (rEAE). A therapeutic treatment model of rEAE in SJL mice. Mice were immunized on day 0 with PLP_139_/complete Freund’s adjuvant (CFA) and pertussis toxin, and their EAE score was monitored from day 7 to the end of the study. Mice were treated once, i.v., on day 14 with empty nanoparticles NP[Empty], nanoparticles containing rapamycin alone (NP[Rapa]) at 5 or 50µg of rapamycin, NP[PLP_139_] at 0.5µg PLP_139_, or tNP at 0.5µg PLP_139_ and 5 or 50µg of rapamycin. **(A)** PLP_139_ and rapamycin contained in tNPs synergize to suppress disease to a greater degree than nanoparticles containing PLP_139_ alone. **(B)** Table of statistical significance from **(A)**. **(C)** tNPs reduce demyelination in the central nervous system compared to NP[Empty] and NP[Rapa]. *N* = 12 for **(A)**. *N* = 6 for **(C)**. Error bars indicate SEM. Statistics are derived from a one-way ANOVA with Tukey Multiple Comparison test. Significance: *p < 0.05; **p < 0.005; ***p < 0.0005; and ****p < 0.0001.

We next assessed the ability of tNPs to therapeutically treat disease in a model of rEAE. In this system, we generated tNPs containing myelin proteolipid protein peptide fragment 139–151 (PLP_139_) with rapamycin or NPs containing PLP_139_ alone (NP[PLP_139_]) for i.v. administration. Across all groups, 5 or 50µg of rapamycin and 0.5µg PLP_139_ were dosed alone or together as NP[PLP_139_], NP[Rapa], or tNP. SJL mice were immunized s.c. with PLP_139_ emulsified in CFA followed by PTx i.p. to induce rEAE. In this model, animals start developing ascending paralysis from day 10 after immunization. In the initial study, mice were administered two doses of NPs on the third day and tenth day after the onset of clinical symptoms. Therapeutic treatment with tNP completely inhibited disease relapse (Figure S3 in Supplementary Material). Next, we evaluated whether a single dose of tNP, administered at the peak of disease, could reverse disease relapse. All animals showed a typical complete remission after the first flare of paralysis with most animals becoming symptomless by day 19. Untreated control animals relapsed 7–8 days after initial disease peak reaching an average EAE disease score of 2 and maintained sickness until the end of the experiment. Relapse was controlled by tNP treatment, as shown by significantly diminished EAE scores compared to all other groups (Figures 4A,B; *p* < 0.0001). The disease was suppressed by tNP for the entirety of the relapsing period, while NP[PLP_139_] reduced the length of relapse and attenuated disease after day 35 (Figures 4A,B). NP[Rapa] did not affect disease relapse at rapamycin doses that matched those administered in the tNP groups. tNP treatment also significantly reduced demyelination in the CNS compared to NP[Empty] and NP[Rapa] (Figure 4C; Figure S2 in Supplementary Material).

### Efficacy of tNP Treatment in an Encephalitogenic T Cell Transfer Model of EAE

**Figure 5:**
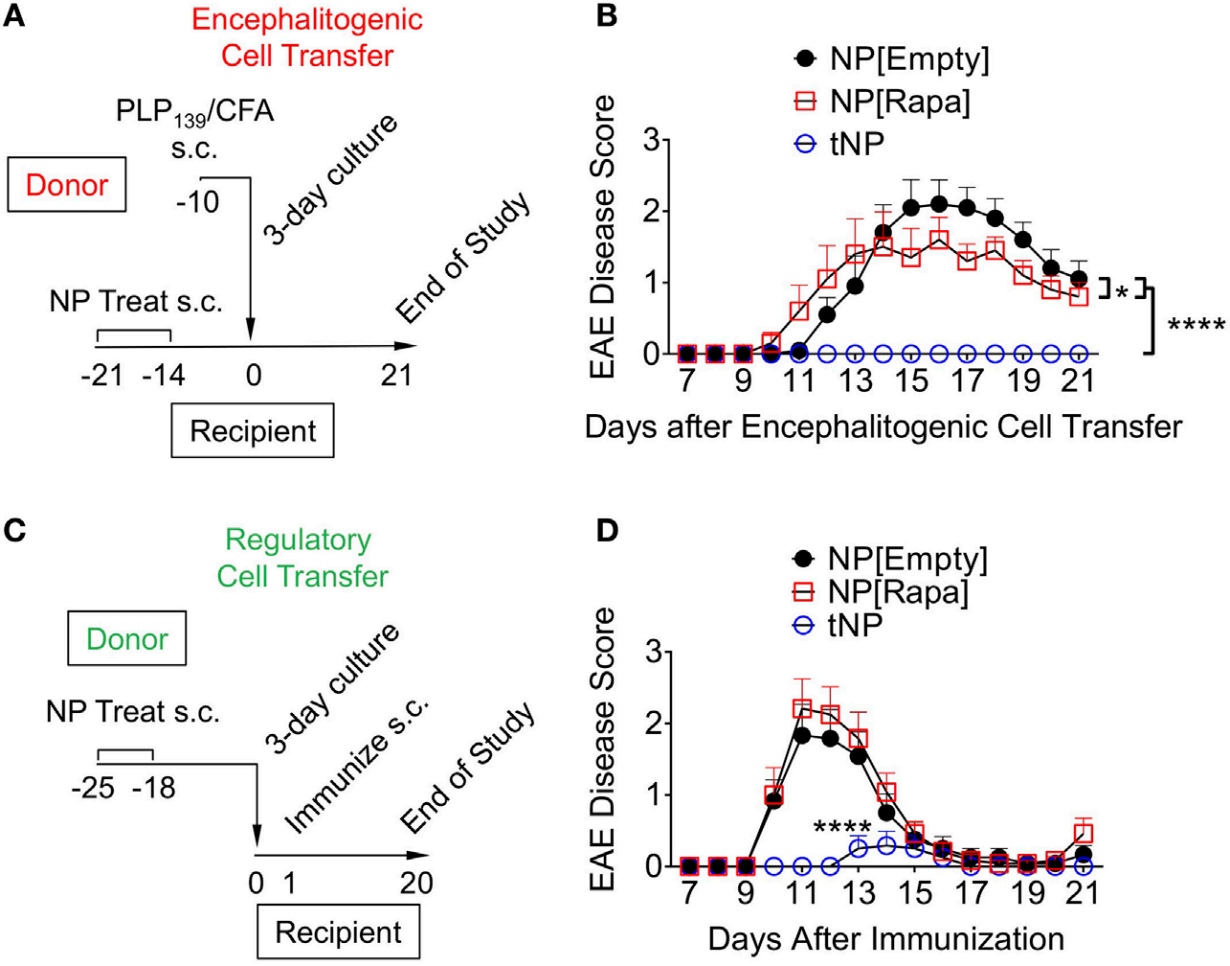
Tolerogenic nanoparticles (tNP) induce prophylactic protection and transferrable tolerance in T cell transfer and direct immunization models of EAE. **(A)** Encephalitogenic cell transfer experimental design. Encephalitogenic cell donors were immunized on day −10, their splenocytes harvested on day −3 and incubated with PLP_139_ for 72 h before transfer into recipients treated on days −21 and −14 with empty nanoparticle (NP[Empty]), nanoparticles containing rapamycin alone (NP[Rapa]) at a 50µg dose of rapamycin, or tNP at a 0.5µg dose of PLP_139_ peptide and 50µg dose of rapamycin. **(B)** EAE scores from **(A)**. **(C)** Regulatory cell transfer experimental design. Donors were treated on days −25 and −18 with NP[Empty], NP[Rapa] at a 50µg dose of rapamycin, or tNP at a 0.5µg dose of PLP_139_ peptide and 50µg dose of rapamycin. Their splenocytes were harvested on day −4 and incubated with PLP_139_ and 100 U/ml of IL-2 for 72 h before transfer into naive recipients. **(D)** EAE scores from **(C)**. The results from **(B,D)** represent an *N* = 12 from one experiment. Error bars indicate SEM. Statistics are derived from a one-way ANOVA with Tukey Multiple Comparison test. Significance: *p < 0.05; **p < 0.005; ***p < 0.0005; ****p < 0.0001.

Encephalitogenic T cells were transferred into recipient mice that had been previously treated with NPs to test whether disease pathogenesis could be contained by an endogenous regulatory response induced by tNPs in the recipient mice. Donor animals were immunized with PLP_139_/CFA on day −10, their spleens were harvested on day −3, and the splenocytes were restimulated *ex vivo* with PLP_139_ and transferred into recipient mice on day 0. The recipients were treated s.c. twice with NP[Empty], NP[Rapa], or tNP on days −14 and −21 prior to cell transfer (Figure 5A). Disease was completely abrogated by tNP prophylaxis, while the NP[Rapa] control had little effect (Figure 5B). These results indicate that treatment with tNPs containing both rapamycin and PLP_139_ induced a durable regulatory response capable of inhibiting pathogenesis mediated by the transferred encephalitogenic T cells.

### tNP Treatment Induces Transferrable Tolerance in a Model of EAE

We next evaluated whether cells from tNP-treated animals could prevent disease in naive recipients immunized with PLP_139_/CFA. Donor mice were treated s.c. with NPs on days −25 and −18. On day −4, their splenocytes were harvested and incubated with PLP_139_ and IL-2 for 72 h, a protocol that has been shown to expand Tregs *in vitro* and used in clinical protocols for Treg immunotherapy of organ transplant and type 1 diabetes (25–28). On day 0, the cells were transferred into naive animals that were then immunized with PLP_139_/CFA (Figure 5C). Transferred splenocytes from tNP-treated mice significantly attenuated and delayed disease compared to splenocytes from both NP[Empty]- and NP[Rapa]-treated donors (Figure 5D). Disease was not suppressed by splenocytes transferred from NP[Rapa]-treated mice. These results indicate that tNP treatment induced a population of regulatory cells capable of conferring protection to naive recipients.

## Discussion

We previously demonstrated that administration of tNP after adoptive transfer of OTII T cells into wild-type mice was capable of inducing OTII Treg and inhibiting their expansion (16, 18). Here, we extend these findings by showing that a single injection of tNP in Rag^−/−^ mice administered 1 day, but not 3 or 5 days, prior to OTII cell transfer into Rag^−/−^ inhibits total Ag-specific T cell proliferation while expanding Ag-specific Tregs (Figures 1A–E). We further demonstrate that tNP treatment increases the proportion and total number of endogenous 2W-specific Foxp3^+^CD4^+^ T cells using 2W:MHCII tetramers in a multiple treatment model (Figures 2D–F), illustrating that tNPs provide a greater tolerogenic stimulus for Foxp3 expression than NP[2W] alone, *in vivo*. This difference was not caused by rapamycin administration alone, as T cells not specific for 2W peptide (2W:MHCII-CD4^+^) did not increase their Foxp3 expression to the same degree as 2W:MHCII^+^CD4^+^ T cells (Figures 2G,H). Upon s.c. challenge with 2W peptide and R848, tNP-treated mice showed fewer IFNγ SFUs from draining LN cells than mice treated with NP[2W] alone or NP[Rapa] alone (Figure 2I). Single (Figures 3A–D) and triple (Figures 3E–H) treatment models show that tNP increased 2W-specific Foxp3^+^CD4^+^ T cells after systemic challenge with 2W peptide co-administered with a potent TLR7/8 agonist or CFA compared to single-component NP controls. In additon, tNPs containing PLP_139_ and rapamycin prevent the pathological impact of encephalitogenic T cell transfer in a model of EAE (Figure 5B). tNPs show greater therapeutic benefit than NP[PLP_139_] in a rEAE model (Figure 4A), while NP[Rapa] alone did not suppress EAE or demyelination at rapamycin doses equal to those in the tNP-treated groups (Figures 4A–C). Finally, EAE was suppressed by adoptive transfer of splenocytes from tNP-treated, but not NP[Rapa]-treated donors (Figure 5D). Together, these results indicate that tNP encapsulating rapamycin with Ag promotes Ag-specific Tregs that persist after Ag challenge in the presence of TLR agonists or CFA. Regulatory cells within the splenic milieu are induced by tNP and capable of transferring tolerance to naive recipients.

The macrolide compound rapamycin is a known inhibitor of mTOR. Previous work has shown the necessity of mTOR signaling to promote T cell expansion (29), differentiation (30), and resistance to anergy (31). The mTOR pathway drives anabolism when activated (32) and autophagy when blocked (33) while continually sensing nutrient levels and stress to confer specific control of those metabolic processes. Pharmacological inhibition of mTOR by chronic dosing with free rapamycin is used to prevent kidney transplant rejection (34). We previously demonstrated that only NP-encapsulated rapamycin, not free rapamycin, is capable of inducing immune tolerance when co-administered with Ag (16). Indeed, while a single dose of tNP containing rapamycin + OVA_323_ peptide inhibited OTII cell expansion, and enhanced the percentage of induced Foxp3^+^ T cells, free rapamycin co-administered with OVA_323_ peptide had the opposite effect, namely enhancing expansion of OTII T cells and reducing the proportion of Foxp3^+^ cells (18). We attribute these findings to tNPs being selectively taken up by APCs in the spleen following i.v. injection, whereas free rapamycin will biodistribute broadly and affect all cell types, including T cells (18).

Antigen-specific therapies for autoimmune diseases would reduce or eliminate the need for chronic immunosuppressant therapy (35). *Ex vivo* expansion of Tregs has been a significant clinical focus to treat autoimmune diseases (36–38); however, current techniques broadly expand polyclonal Tregs, not just Ag-specific cells, require personalized therapies that involve costly and complex manufacturing processes and carry the risk of expanding “unstable” Tregs that lose their regulatory function and can exacerbate disease. “Off the shelf” approaches include strategies to induce tolerogenic DC subsets *in vivo*, which can induce and expand Ag-specific Tregs (13, 39–42). Free peptide Ags may directly bind cell surface MHCII without processing and promote T cell anergy by presenting Ag in the absence of a co-stimulatory signal. Ag can also be targeted directly to APCs through antibody fusion proteins, such as anti-DEC205-PLP_139_ (43), or indirectly by targeting apoptotic red blood cells (44).

Synthetic NPs are an attractive strategy to target DCs and other APCs as these cells are very efficacious at capturing nanoparticulates. NPs have been shown to selectively traffic and accumulate in lymphoid tissues, such as lymph nodes following s.c. injection and the spleen and liver following i.v. injection, where they are selectively endocytosed by Ag-presenting cells (18, 45, 46).

Nanoparticles carrying peptides in the absence of an immunomodulator have been shown to be protective in EAE (47, 48) by targeting MARCO^+^ macrophages or liver sinusoidal cells. A potential concern is that endocytosis of NPs containing Ag alone by activated APCs in an inflammatory microenvironment could present the Ag in a stimulatory context and inadvertently exacerbate disease. Moreover, this approach is limited to peptide Ags, as protein Ags encapsulated in particles are likely to be immunogenic. In our hands, NPs encapsulating peptide alone showed efficacy in EAE, but the protection was incomplete compared to tNP containing both rapamycin and peptide Ag (Figure 4A). Differences in NP size and surface properties may target different populations of cells (49).

Nanoparticles can be engineered to carry an immunomodulator payload that forces DCs to present Ag in a tolerogenic manner, even in a pro-inflammatory environment. Importantly, tNP containing Ag and rapamycin induced Treg populations that were maintained even after Ag challenge administered with a potent TLR agonist (Figures 3C,D). Moreover, tNPs were equally effective with both peptide and protein Ag to prevent antibody responses and have shown therapeutic efficacy in EAE following s.c. or i.v. administration [Figure 4 (18)]. Therapeutic efficacy has also been shown in EAE with polyclonal expansion of Tregs from spleen and CNS after intranodal injection of microparticles encapsulating peptide and rapamycin (20). In addition to rapamycin, NPs delivering Ag with other immunomodulators, such as aryl hydrocarbon receptor ligands (50) and NFκB inhibitors (51), have also been shown to be effective in treating animal models of autoimmune disease.

While many promising preclinical approaches for Ag-specific immune tolerance have been described, few have reached clinical trials, and even fewer have shown evidence of efficacy in humans. There are several fundamental challenges in translating data from mice to humans; (1) selection of the appropriate Ag, (2) human genetic variation, and (3) achieving therapeutic efficacy in a well-established disease. Ag selection is simple in contrived animal models such as EAE where disease is induced by immunization with a specific Ag. In some diseases, candidate Ags have been identified; however, the specific pathogenic Ags may vary from patient to patient and evolve through epitope spreading. Peptide-specific approaches are relatively straightforward using inbred strains of mice. However, MHC heterogeneity in humans poses a challenge to create a manageable set of peptides providing coverage for all major Ags for all patients. Finally, it is difficult to assess efficacy of therapeutic candidates in well-established disease in mice due to their short lifespan and the limitations of available models. Clinical trials with a cocktail of free peptide Ags (52) or peptides conjugated to autologous leukocytes (53) have reported initial biomarker evidence for Ag-specific immune modulation. Further studies are required to determine the level of efficacy and durability of therapy.

To mitigate some of the difficulties in establishing and evaluating immune tolerance induction in humans, we have chosen to focus initially on mitigation of antidrug antibodies (ADAs) to biologic therapies. The advantages from a drug development stand point are the elimination of Ag risk, as the Ag is the biologic drug, the ability to first assess tolerance in a prophylactic treatment setting and clear biomarker readout (i.e., ADAs). We have demonstrated the ability of NPs encapsulating rapamycin to inhibit the formation of ADAs against a variety of biologic drugs in preclinical studies, including coagulation factor VIII in hemophilia A mice (19), myozyme (or acid alpha glucosidase) in a murine model of Pompe disease (17), humira in a spontaneous model of inflammatory arthritis, and pegylated uricase enzyme in both Urox-deficient mice and non-human primates (16). The safety and efficacy of SEL-212, a combination of NP-encapsulated rapamycin co-administered with pegylated uricase, is currently being evaluated in an ongoing multidose Phase 2 clinical study in symptomatic gout patients with hyperuricemia (NCT02959918). Initial data from the single ascending dose Phase I clinical trial of SEL-212 (NCT02648269) showed dose-dependent inhibition of anti-uricase antibodies with a corresponding sustained reduction of serum uric acid (54). The ongoing Phase 2 study will assess the ability of tNPs to induce immune tolerance in patients.

## Ethics Statement

Experiments involving animals were performed in compliance with state and federal regulations and approved by the Institutional Animal Care and Use Committee (IACUC) of Selecta Biosciences or Hooke Laboratories.

## Author Contributions

RL, RM, JF, and TK designed and executed the experiments. RM and TK reviewed and edited the manuscript. TV and TG formulated the nanoparticles. VC, PD, AS, and SA analyzed the small molecule and peptide content of the nanoparticles. RL performed OTII and 2W cell analysis assays. PK and JW performed IFNγ ELISpots. Hooke Laboratories performed the rEAE experiments. RL wrote the manuscript.

## Conflict of Interest Statement

All authors are current or former employees and/or shareholders of Selecta Biosciences. The reviewer AM and handling editor declared their shared affiliation.
