# Supplementary figures and images for: Tolerogenic Nanoparticles Induce Antigen-Specific Regulatory T Cells and Provide Therapeutic Efficacy and Transferrable Tolerance against Experimental Autoimmune Encephalomyelitis
Source: Front Immunol. 2018 Mar 2;9:281. doi: 10.3389/fimmu.2018.00281 (PMC5840162; doi:10.3389/fimmu.2018.00281)

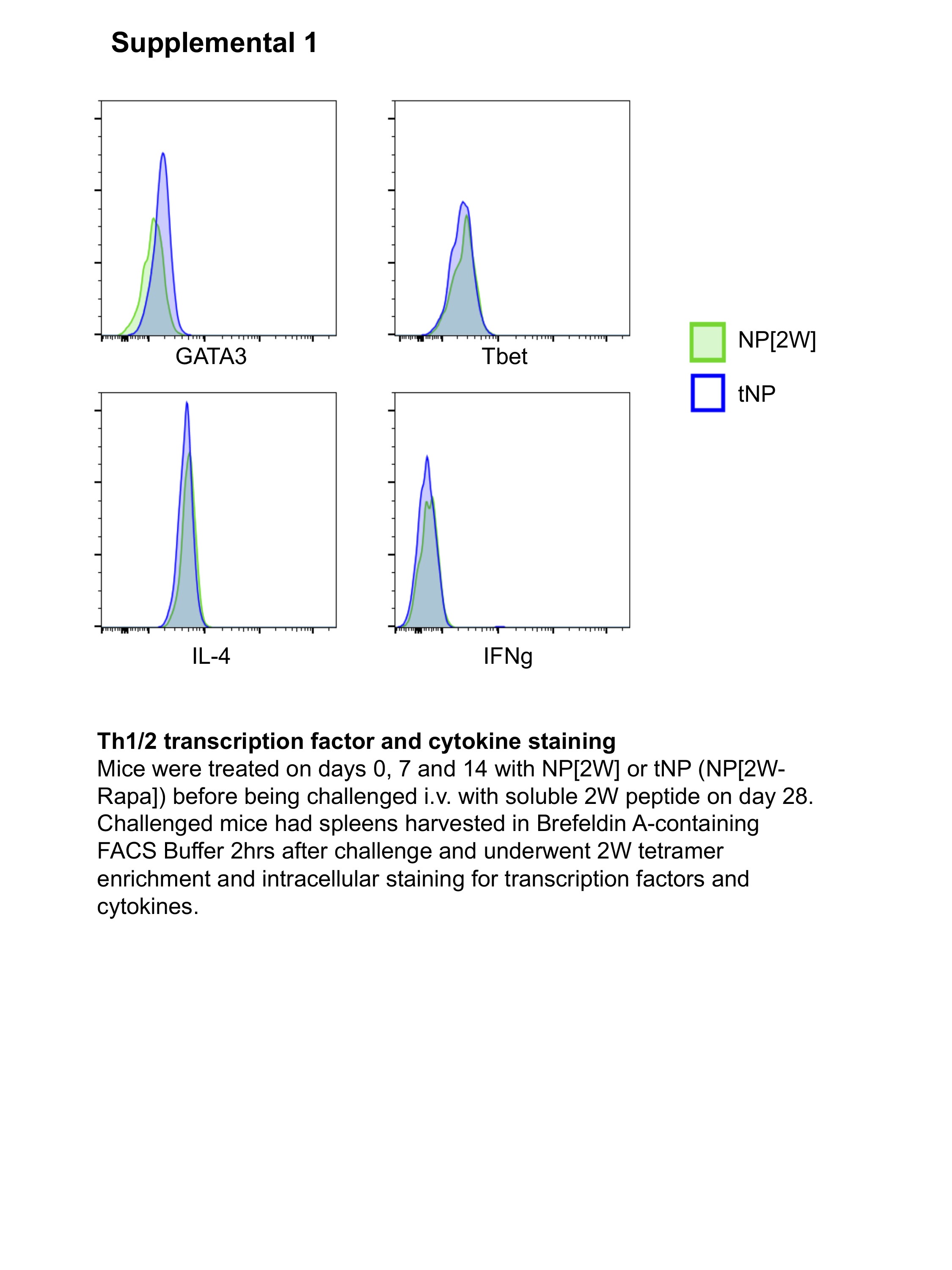

Supplement: Supplementary file 1 [file Image_1.jpg]

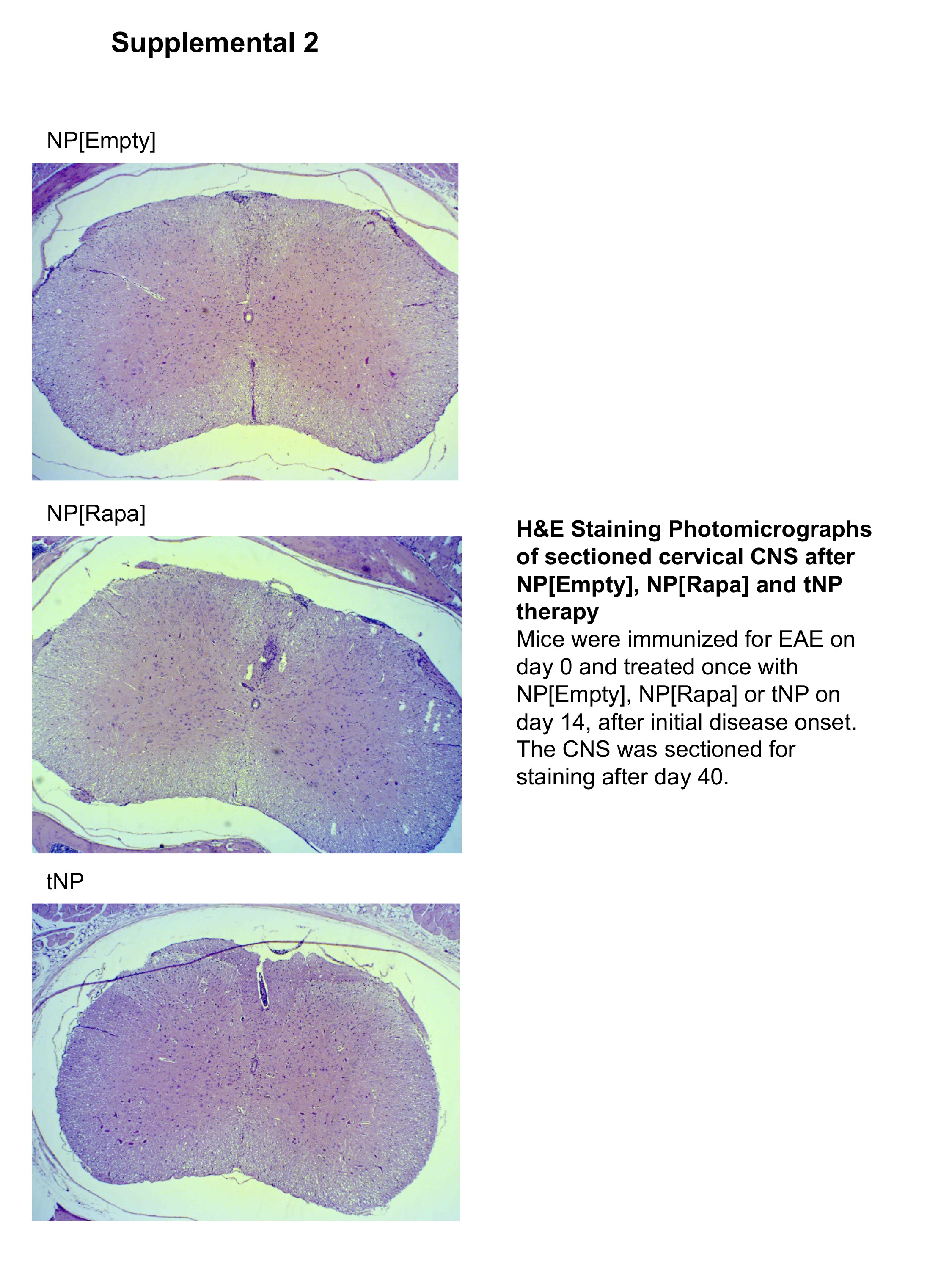

Supplement: Supplementary file 2 [file Image_2.jpg]

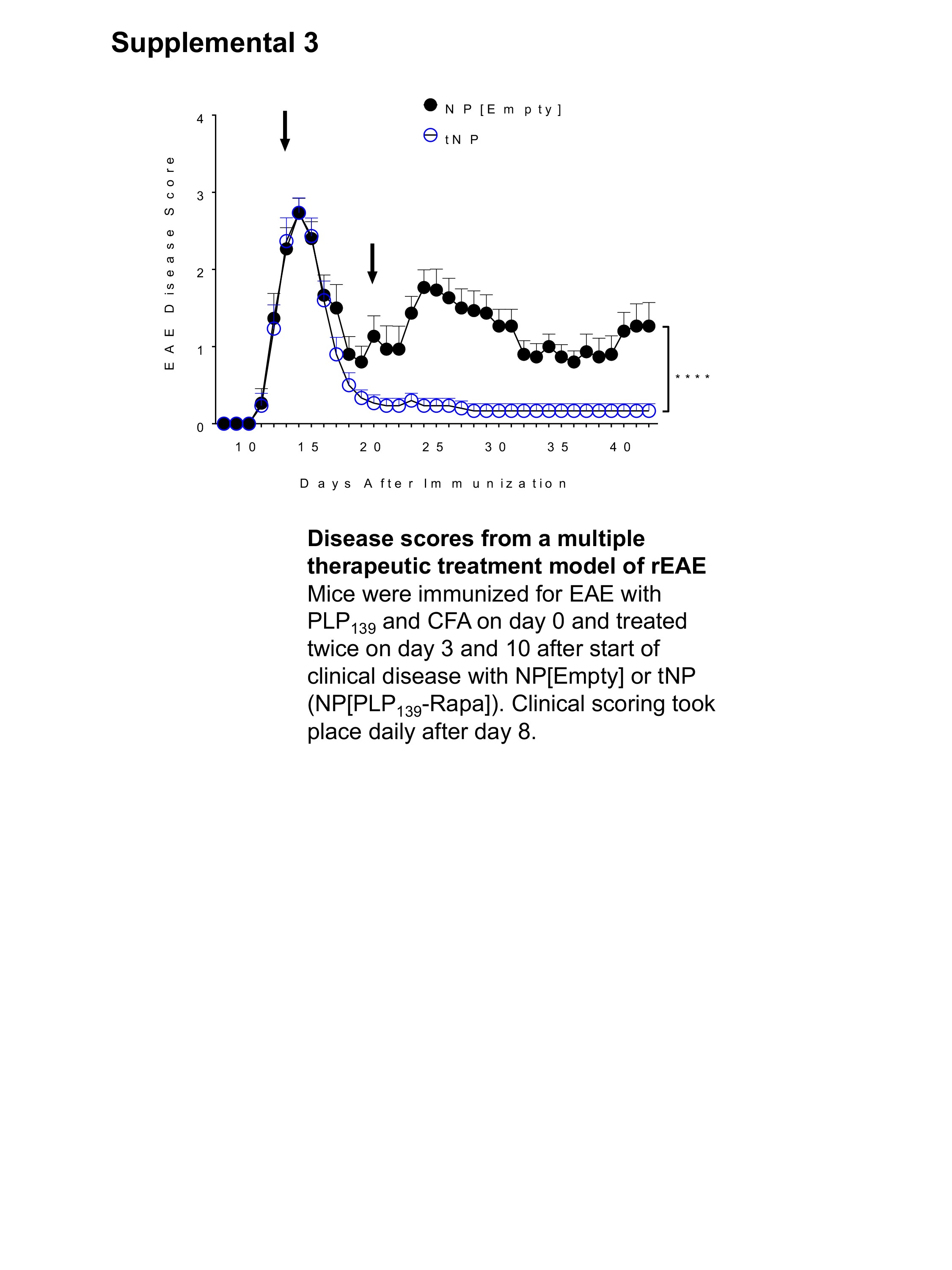

Supplement: Supplementary file 3 [file Image_3.jpg]
